# Probabilistic Record Linkage for Families (PRLF): A Discussion of the Development and Validation of this Open-Source Linkage Tool

**DOI:** 10.23889/ijpds.v9i5.2765

**Published:** 2024-09-18

**Authors:** John Prindle, Himal Suthar, Emily Putnam-Hornstein, Regan Foust

**Affiliations:** 1 USC Suzanne Dworak-Peck School of Social Work; 2 UNC School of Social Work

## Objectives

Synthetic data (SD) promises to unlock health data for training, research, and innovation. However, where utility evaluation is performed, it is applied ad-hoc for a single task of interest. We produce an initial design for a robust benchmark across a range of tasks.

## Approach

We undertook several projects as a prototyping experiment to gather requirements. These projects replicate previous studies performed on the Medical Information Mart for Intensive Care — a dataset used in more than 4,000 studies. We refine definitions, identify personas, draft a user statement, and collect requirements.

## Results

Definitions: We define utility as an extrinsic measure of SD on a larger system, most often through comparison to system performance on real data. This contrasts with fidelity, which measures the accuracy of SD through direct comparison to real data.

Personas: Data custodian, User of SD, SD researcher.

User statement: As a technical stakeholder, I need a reliable way to measure the utility of datasets and a benchmark to compare generation techniques.

Requirements: SD researchers can focus on generation not evaluation; Supports comparison and leaderboards; Based on relevant and applications; Comprehensive across study types and applications; Future proof for population research requiring linking.

## Conclusion

We propose the following design:

- Data pipelines follow an extract-generate-evaluate workflow.
- Study types include cross-sectional and longitudinal.
- Applications include predictive modelling and clinical research.

This results in a comprehensive utility benchmarking suite that complements current frameworks for fidelity and privacy of SD.

